# Sex and ethnic/racial differences in disordered eating behaviors and intuitive eating among college student

**DOI:** 10.3389/fpsyg.2023.1221816

**Published:** 2023-09-18

**Authors:** Cynthia Yoon, Dan Mai, Kush Kinariwala, Tracey Ledoux, Randi Betts, Craig Johnston

**Affiliations:** Department of Health and Human Performance, University of Houston, Houston, TX, United States

**Keywords:** disordered eating behaviors, intuitive eating, college students, cross-sectional, sex, ethnicity, race

## Abstract

**Introduction:**

Eating behaviors encompass disordered eating behaviors (e.g., overeating, binge eating, and associated symptoms of binge eating) and intuitive eating. Certain disordered eating behaviors, including binge eating, are more prevalent among female and ethnic/racial-minority college students than male and/or non-Hispanic White college students. However, sex and ethnic/racial differences among college students with other disordered eating (e.g., associated symptoms of binge eating) and intuitive eating behaviors remain unclear.

**Methods:**

In 2022, 887 college students (*M*_age_ = 20.9 ± 2.6 years) self-reported their sex, ethnicity/race, disordered eating behaviors (e.g., overeating, binge eating, associated symptoms of binge eating), and intuitive eating. To examine sex and ethnic/racial differences among these students, we used modified Poisson regressions for students who reported disordered eating and linear regressions for students who reported intuitive eating.

**Results:**

Except for overeating, disordered eating behaviors were more prevalent among female [adjusted prevalence ratio (aPR) = 1.3–1.8] than male college students after adjusting for sociodemographic variables, whereas intuitive eating scores did not differ by sex. Across ethnic/racial groups, disordered eating was more prevalent among all ethnic/racial-minority college students (aPR = 1.2–2.3) than non-Hispanic White college students after adjusting for sociodemographic variables. Moreover, non-Hispanic Black or African American college students had higher intuitive eating scores than non-Hispanic White college students (adjusted β = 0.7, 95% CI = −0.2, 1.6).

**Conclusion:**

In our sample, notable differences emerged in the prevalence of disordered eating behaviors and mean scores by sex and ethnicity/race, while differences in intuitive eating scores emerged based on ethnicity/race.

## Introduction

Eating behaviors encompass disordered eating behaviors and intuitive eating. Examples of disordered eating behaviors include binge eating and unhealthy weight control behaviors (Cain et al., 2012; Arigo et al., 2014; Nagata et al., 2018; Neumark-Sztainer et al., 2018). Disordered eating behaviors are concerning given their persistence over time (Neumark-Sztainer et al., 2018; Stok et al., 2018) and association with adverse health outcomes—for example, excessive weight gain and an increased risk of clinically diagnosable eating disorders (Sonneville et al., 2013; Mustelin et al., 2017; Kärkkäinen et al., 2018; Yoon, et al., 2020a). In contrast, intuitive eating (Tylka and Wood-Barcalow, 2015) represents a nondieting approach that rejects reliance on external factors; instead, it involves attuning oneself to the body’s internal cues, including hunger, fullness, and satisfaction, to guide eating habits. Intuitive eating is commonly assessed using the intuitive eating scale, which comprises four subscales: unconditional permission to eat, eating for physical rather than emotional reasons, reliance on hunger and satiety cues, and body-food choice congruence (Tylka and Kroon Van Diest, 2013). Of them, the reliance on hunger and satiety cues subscale specifically emphasizes the importance of recognizing hunger and fullness by eating when physically hungry and stopping eating when physically full. Individuals who place greater reliance on those internal cues than external cues tend to experience improved health outcomes, including more effective weight management, a reduced risk of engaging in disordered eating behaviors, and enhanced psychological well-being (Denny et al., 2013; Hazzard et al., 2020; Smith et al., 2020; Tylka et al., 2020; Christoph et al., 2021a,b; Gödde et al., 2022).

Given the consequences of disordered eating behaviors and the reliance on hunger and satiety cues in intuitive eating, it is imperative to identify individuals at risk of disordered eating and those who score low on the reliance on hunger and satiety cues subscale of the intuitive eating scale. Although both males and females are susceptible to disordered eating behaviors and low scores for intuitive eating, studies to date have been conducted primarily among females (Hawks et al., 2005; Avalos and Tylka, 2006; Kelly-Weeder, 2012; Arigo et al., 2014; Andrew et al., 2015; Arroyo et al., 2017), albeit with a growing body of literature examining eating behaviors exclusively among men (Lavender et al., 2010, 2017; Cain et al., 2012) or among both males and females (Gast et al., 2012; Denny et al., 2013; Mustelin et al., 2017; Kärkkäinen et al., 2018; Nagata et al., 2018; Neumark-Sztainer et al., 2018; Horwath et al., 2019; Tylka et al., 2020; Yoon et al., 2020a).

In particular, studies examining sex differences in disordered eating behaviors have been limited to binge eating and unhealthy weight control behaviors (e.g., fasting and use of diet pills) (Striegel-Moore et al., 2009; Lavender et al., 2010; Cain et al., 2012; Arigo et al., 2014; Nagata et al., 2018; Neumark-Sztainer et al., 2018). Although binge eating is frequently accompanied by various eating behaviors, including eating until uncomfortably full, eating quickly, eating alone due to embarrassment, and experiencing feelings of disgust and depression afterward, all hereafter referred to as “associated symptoms of binge eating” (Lee-Winn et al., 2014), very few studies have investigated sex differences in those associated symptoms of binge eating (Lee-Winn et al., 2014). Although a study has shown that associated symptoms of binge eating are more prevalent among middle-aged females than males, those findings have not been replicated within other age groups to confirm the robustness and generalizability of the findings.

Research has further indicated that the prevalence of concerns about weight and body shape may vary according to sex, given differences in socially acceptable ideals of body image. For example, thinness is more widely promoted and idealized among females than males, hence females’ relative interest in reducing their body size (Myers and Crowther, 2007). In contrast, males may face sociocultural pressure to attain a lean, muscular physique, hence their relative interest in increasing their body size (Lavender et al., 2017; Kukk and Akkermann, 2020; Schaefer et al., 2021). Together, these findings highlight the importance of investigating sex differences in relation to associated symptoms of binge eating and concerns regarding weight and body shape.

Aside from disordered eating, a growing body of studies have examined sex differences in intuitive eating. In samples of young adults, males have consistently shown higher mean scores than females (Denny et al., 2013; Smith et al., 2020). In particular, research conducted in the United States and Germany has shown that males tend to score higher than females on the reliance on hunger and satiety cues subscale (van Dyck et al., 2016; Rodgers et al., 2021). Even so, no significant sex difference in scores on the subscale has been found in a general sample of adults in France (Camilleri et al., 2015). Given those inconclusive findings and limited research using the reliance on hunger and satiety cues subscale, additional studies are needed to explore sex differences in the reliance on hunger and satiety cues. Moreover, assessing potential sex differences in associated symptoms of binge eating and concerns about weight and body shape could afford more comprehensive insights for understanding intuitive eating overall, including by using the reliance on hunger and satiety cues subscale.

Along with exploring sex differences in disordered eating behaviors and intuitive eating, studies have also documented the need to examine ethnic/racial differences in such eating behaviors, owing to different cultural norms and societal expectations that may affect ideal body image. Traditionally, non-Hispanic whites have been shown to idealize lean bodies (Gillen and Lefkowitz, 2012), whereas African Americans have been shown to value larger body frames (Awad et al., 2015). Moreover, whereas Hispanic culture often emphasizes fuller figures as being desirable (Lezama and Castillo, 2020), Asian culture shows a preference for slim, lean figures (Brady et al., 2017). These cultural variations in body ideals underscore the need to consider ethnic/racial differences to better understand and address eating behaviors in general. Although ethnic/racial differences in body image have prompted studies on such differences in relation to disordered eating behaviors, their findings have not revealed any consistent patterns. For instance, several studies have demonstrated that binge eating is more prevalent among Black or non-Hispanic African Americans (Marques et al., 2011; Simone et al., 2022), Hispanics (Marques et al., 2011; Simone et al., 2022), and non-Hispanic Asians (Lee-Winn et al., 2014) than non-Hispanic White individuals; however, other studies have shown no significant ethnic/racial differences in disordered eating behaviors (Lee-Winn et al., 2016). Apart from binge eating, a nationally representative study limited to Asian Americans and non-Hispanic Whites investigated associated symptoms of binge eating (e.g., eating alone and feeling distressed after eating) and revealed notable ethnic/racial differences, including a lower prevalence of those eating behaviors among Asian Americans than non-Hispanic Whites (Lee-Winn et al., 2014). However, because the study sample was limited to Asian Americans and non-Hispanic Whites, research replicating the study with ethnically/racially diverse samples is needed to enhance the generalizability of the results. Additional research is also needed to clarify potential variations in associated symptoms of binge eating across different ethnic/racial groups.

In the increasing body of literature addressing ethnic/racial differences in intuitive eating, one study has revealed that African Americans and Hispanics were more likely to engage in intuitive eating than non-Hispanic White individuals (Denny et al., 2013). Conversely, another study showed no significant differences in intuitive eating scores across ethnic/racial groups (Gödde et al., 2022). Among young adults, another study revealed that Native Americans were more likely to stop eating when full than other ethnic/racial groups (Denny et al., 2013). However, to our knowledge, only one study has examined reliance on hunger and satiety cues subscale of intuitive eating across six ethnic/racial groups; among its findings, African Americans had higher scores for reliance on hunger and satiety cues than any other ethnic/racial group (Tylka, 2006). On the whole, the limited but growing body of literature focused on ethnic/racial differences in intuitive eating highlights the need for replication studies using ethnically/racially diverse samples.

Considering that eating disorder onset tends to peak during young adulthood, the majority of studies examining eating behaviors have focused on either adolescents or young adults (Hudson et al., 2007; Kessler et al., 2013; Solmi et al., 2022). However, college students, ranging from 17 to 24 years of age, represent a unique population of emerging adults who are transitioning from adolescence to adulthood (Nelson et al., 2008). That transitional period coincides with the age range when eating disorder onset tends to peak (Hudson et al., 2007; Kessler et al., 2013; Solmi et al., 2022), thereby emphasizing the need for increased research targeting college students. Indeed, studies have consistently demonstrated that a substantial proportion of college students, up to two-thirds, engage in various forms of disordered eating behaviors, including dieting, binge eating, or purging behaviors (White et al., 2011; Kelly-Weeder, 2012). Thus, there is a compelling rationale for examining sex and ethnic/racial differences in eating behaviors within the college student population to better understand and address those concerns.

The inconclusive findings on sex and ethnic/racial differences in disordered eating and the scarcity of studies examining those differences in intuitive eating underscore the need for studies that comprehensively explore sociodemographic differences within a broad range of eating behaviors in diverse samples of college students. Such findings may provide insights into who is more likely to engage in disordered eating and whether a sex- and/or ethnic/racial- inclusive approach is needed to prevent disordered eating and support intuitive eating. Understanding sociodemographic differences in eating behaviors may have clinical implications for screening and prevention efforts and may enable the development of culturally appropriate health programs to tailor interventions and thereby reduce potential health disparities on college campuses.

To this end, this study aims to assess disordered eating behaviors (e.g., binge eating, unhealthy weight control behaviors, and concerns with weight and body shape) and intuitive eating among college students. To examine sex and ethnicity/race disparities in a broad range of eating behaviors among college students, this study aims to examine how such eating behaviors differ by sex and ethnicity/race in college students. Drawing from past studies (Kelly-Weeder, 2012; Denny et al., 2013; Lee-Winn et al., 2016; Lipson and Sonneville, 2017; Smith et al., 2020) and theories (Meyer, 2003; Niva, 2017), it is hypothesized that disordered eating will be more prevalent and that intuitive eating scores will be lower among female and ethnic/racial minority college students than among non-Hispanic White college students.

## Materials and methods

### Design

The study sample included both male and nonpregnant female undergraduate students at least 18 years old who were enrolled in an introductory course in kinesiology during the Spring 2022 semester at a university in Texas. The course, Introduction to Kinesiology, is part of the Texas core curriculum in the area of the social and behavioral sciences. Open to students in any major, the course does not require any prerequisites and is designed to provide an introduction to kinesiology for all undergraduate students. Thus, students in the course have diverse academic backgrounds and are at different stages of their undergraduate education.

Once the study’s protocol was reviewed and approved by the Institutional Review Board, the course’s instructor invited students to participate in the study, described as having the purpose of examining college students’ eating behaviors. The invitation was issued on the Blackboard learning management system. Students interested in the study provided their written informed consent to participate, were shown a URL to access and complete an online survey and were subsequently directed to schedule an anthropometric assessment in the research lab. The survey took approximately 30 min to complete and was available for approximately 8 weeks to accommodate students’ response times. Completing both the survey and the anthropometric measurements was required for complete participation in the study. For an incentive, participants who completed the study were either awarded course credit or entered into a raffle for $25 gift cards.

### Data collection

Data were collected with the survey and anthropometric measurements. First, students were asked to complete the abovementioned online survey, administered via a questionnaire on Qualtrics.^1^ Participants were informed of their right to withdraw from the survey at any time and were given the response option of “prefer not to answer” for all questionnaire items. Prior to being administered, the questionnaire underwent pilot testing with five research staff members at the university. The online survey included questions related to participants’ eating behaviors, past life experiences, and sociodemographic variables.

Second, the participants’ anthropometric measures were taken by trained research assistants. Weight and body mass index (BMI) were measured using a Tanita Bioelectrical Impedance Analyzer DC-430 (Tanita Corporation, Chicago, IL, USA). Prior to measurement, each participant’s height, age, and sex were entered into the device. During measurement, participants stood barefoot on the device’s footpads and ensured that both feet touched the electrodes. To minimize the impact of dehydration or overhydration on the measurements taken, participants were instructed to have an empty bladder and to avoid engaging in intense exercise before visiting the lab. Next, height was measured using a Seca 222 Height Measuring Stadiometer (Quick Medical, Issaquah, WA, USA) with each participant standing erect without shoes. Body mass index (BMI) was calculated using the height and weight measurements obtained.

### Participants

Of the 1,185 students invited to participate in the study, 983 (82.3%) participated. However, participants with missing information about their eating behaviors (*n* = 33), sex (*n* = 29), ethnicity/race (*n* = 11), or covariates (*n* = 22) were further excluded. The final sample for analysis contained 887 participants, or 74.9% of the 1,185 students invited and 90.2% of the 983 participants. Participants and nonparticipants did not differ significantly in demographic characteristics—that is, sex, ethnicity/race, and academic standing (*p* ≥ 0.05).

### Measures

#### Independent variables

##### Sex

Sex assigned at birth was self-reported and categorized into “male” or “female.”

##### Ethnicity/race

Ethnicity/race was self-reported and assessed using two items: “What is your ethnicity?” and “What is your racial identity?” Ethnicity/race was categorized as (a) non-Hispanic White, (b) non-Hispanic Black or African American, (c) Hispanic, (d) non-Hispanic Asian, or (e) racial minority or other (i.e., American Indian, Alaskan Native, Native Hawaiian, Pacific Islander, or multiple racial identities).

#### Dependent variables

##### Disordered eating

Items from the Questionnaire on Eating and Weight Patterns-5 (Yanovski et al., 2015) were adapted to assess five disordered eating behaviors in this study: overeating, binge eating, unhealthy weight control behaviors, associated symptoms of binge eating, and concerns about weight and body shape. The five disordered eating behaviors assessed in this study have been examined in prior research and demonstrated predictive validity for various health outcomes (Yoon et al., 2018, 2019, 2022c). These five disordered eating behaviors have further been supported by their coherence as a set of indicators of disordered eating behaviors and have been used to construct a comprehensive scale to measure the severity of disordered eating attitudes and behaviors (Yoon et al., 2020b).

For items related to overeating, binge eating, unhealthy weight control behaviors, and associated symptoms of binge eating, participants were provided with response options of “yes” and “no” to indicate their engagement in those behaviors. Regarding concerns about weight and body shape, participants were asked to indicate their level of concern by selecting one of the following statements: “Weight and shape are not very important,” “Weight and shape play a part in how I feel about myself,” “Weight and shape are among the main things that affect how I feel about myself,” or “Weight and shape are the most important things that affect how I feel about myself.” To distinguish participants with concerns about their weight and body shape, the cutoff points recommended in the *Diagnostic and Statistical Manual of Mental Disorders, Fifth Edition* (*DSM-5*) were used [American Psychiatric Association (APA), 2013]. Specifically, participants who indicated that weight and body shape were among or the most important things affecting how they felt about themselves were categorized as having concerns with weight or body shape. Although the timeframe for overeating and binge eating in the DSM-5 refers to the past 6 months, the timeframe in this study referred to the past year, given that the aim of this study was to capture subclinical disordered eating behaviors, not to clinically diagnose eating disorders. Past studies examining disordered eating behaviors have also used the time frame of the past year to capture overeating and binge eating (Yoon et al., 2021, 2022a,b). Cronbach’s alpha for the questionnaire items in this sample was 0.75, which indicates acceptable reliability.

##### Intuitive eating

Six items were adapted from the reliance on hunger and satiety cues subscale of the Intuitive Eating Scale-2 (Tylka and Kroon Van Diest, 2013), including “I trust my body to tell me when to eat” and “I rely on my fullness (satiety) signals to tell me when to stop eating.” A summary score for reliance on hunger and satiety cues in intuitive eating was created and ranged from 6 to 24 points, with higher scores indicating higher levels of reliance on hunger and satiety cues in intuitive eating. Cronbach’s alpha for the reliance on hunger and satiety cues in intuitive eating in this sample was 0.83, which indicates good reliability.

### Covariates

Variables associated with disordered eating behaviors and having the potential to introduce biases were selected as confounders (Wang et al., 2017). In the models examining the associations of eating behaviors with sex, the variables of age, ethnicity/race, either parent’s highest level of education and BMI were considered potential confounders. In contrast, in the models examining the associations of ethnicity/race with eating behaviors, the variables of age, sex, either parent’s highest level of education, and BMI were considered potential confounders. Parental education was considered a potential confounder based on a study suggesting that parental education, income, and occupation serve as proxies for socioeconomic status (Oakes and Rossi, 2003). Among these three proxies, parental education has been most robustly associated with physical and mental health outcomes (Nagata et al., 2018). For these reasons, in epidemiological studies of young adults (Araya et al., 2003; Geyer et al., 2006; Wang et al., 2009; Hudson et al., 2013), as well as in studies of eating disorders (Hay et al., 2015; Tabler and Utz, 2015), parental education is regarded as the standard socioeconomic proxy. Additionally, given extant studies that document adults with low socioeconomic status tend to have a higher BMI (Newton et al., 2017) and that a higher BMI is linked to disordered eating due to the impact of weight stigma (Nagata et al., 2018), BMI was also considered as a potential confounder. Models adjusted for these confounders present individuals’ positions within race, sex, and class-based social hierarchies, which significantly influence individual vulnerabilities to eating behaviors (Weinstein et al., 2017).”

### Statistical analysis

Power calculation was not performed because our study was exploratory in nature—that is, conducted to inform the design of future studies. In this study, participants’ characteristics are presented as the means (*SD*) or frequency (%). The percentages of college students reporting eating behaviors were examined across sex and ethnicity/race, and chi-square values are reported. Modified Poisson regressions were used to model the relationship of disordered eating as a function of sex and ethnicity/race. In the results, the prevalence ratio (PR) and 95% confidence intervals (CIs) are provided with the null value of 1.0. Linear regressions were used to model the relationship of reliance on hunger and satiety intuitive eating as a function of sex and ethnicity/race. Beta coefficients and 95% CIs are provided with a null value of 0.0. Models were adjusted for sociodemographic characteristics. All statistical analyses were conducted using SAS 9.4 (SAS Institute Inc., Cary, NC) (SAS Institute Inc, 2016).

## Results

### Sample characteristics

The participants included 887 college students in Texas with a mean age of 20.9 ± 2.6 yrs. Among the 887 participants, 54.2% were females; 15.1% were non-Hispanic White, 14.1% were non-Hispanic Black or African American, 33.2% were Hispanic, 35.0% were non-Hispanic Asian, and 2.7% were racial minority or other (i.e., American Indian, Alaskan Native, Native Hawaiian, Pacific Islander, or Multiracial) (Table 1).

**Table 1:** Sociodemographic characteristics of the participants (*N* = 887).

	Number of participants (*N* = 887)
Age *M* (years) ± SD	20.9 ± 2.6
Sex *n* (%)	
Female	481 (54.2)
Male	406 (45.8)
Ethnicity/Race *n* (%)	
Non-Hispanic White	134 (15.1)
Non-Hispanic Black or African American	125 (14.1)
Hispanic	294 (33.2)
Non-Hispanic Asian	310 (35)
Racial minority or other	24 (2.7)
Parental education attainment *n* (%)	
Less than high school	82 (9.2)
High school completion or more	131 (14.8)
Partial college degree	157 (17.7)
Bachelor’s degree or more	312 (35.2)
Master’s degree or more	205 (23.1)

Racial minority or others includes American Indian, Alaskan Native, Native Hawaiian, Pacific Islanders.

### Sex differences in eating behaviors

#### Disordered eating behaviors

The prevalence of disordered eating behaviors for both male and female college students is reported in Appendix Table 1. The prevalence of overeating among female college students was 20% lower than that among male college students after adjustment for age, ethnicity/race, parental maximum educational attainment, and BMI (aPR = 0.8, 95% CI = 0.7–1.0). Female college students had 1.3 to 1.8 times the prevalence of all other disordered eating behaviors compared to male college students [aPR = 1.8, (95% CI = 1.4–2.3), aPR = 1.3 (95% CI = 1.2–1.5), aPR = 1.5 (95% CI = 1.2–1.9) and aPR = 1.3 (95% CI = 1.1–1.5) for binge eating, unhealthy weight control behaviors, associated symptoms of binge eating, and concerns with weight and body shape, respectively] after adjustment for sociodemographic variables and BMI (Table 2).

**Table 2:** Prevalence ratios of disordered eating and mean intuitive eating score differences by sex and ethnicity/race (*N* = 887).

	Sex		Ethnicity/race				
	Male (*n* = 406)	Female (*n* = 481)	Non-Hispanic White (*n* = 134)	Non-Hispanic Black or African American (*n* = 125)	Hispanic (*n* = 294)	Non-Hispanic Asian (*n* = 310)	Racial- minority or other (*n* = 24)
Disordered eating behaviors aPR^a^ (95% CI)							
Overeating^b^	1.00 (ref)	0.8 (0.7–1.0)	1.00 (ref)	1.1 (0.8–1.3)	0.9 (0.7–1.1)	1.2 (0.8–1.7)	0.9 (0.7–1.3)
Binge eating^c^	1.00 (ref)	1.8 (1.4–2.3)	1.00 (ref)	1.1 (0.7–1.7)	1.3 (0.9–2.0)	0.8 (0.3–2.0)	1.2 (0.8–1.9)
Unhealthy weight control behaviors^d^	1.00 (ref)	1.3 (1.2–1.5)	1.00 (ref)	1.1 (0.9–1.3)	1.2 (1.0–1.5)	1.1 (0.7–1.7)	1.0 (0.7–1.2)
Associated symptoms of binge eating^e^	1.00 (ref)	1.5 (1.2–1.9)	1.00 (ref)	2.3 (1.4–3.5)	2.1 (1.4–3.3)	1.4 (0.6–3.4)	2.1 (1.3–3.4)
Concerns with weight and body shape^f^	1.00 (ref)	1.3 (1.1–1.5)	1.00 (ref)	1.5 (1.1–1.9)	1.4 (1.1–1.8)	1.2 (0.7–2.1)	1.2 (0.9–1.7)
Intuitive eating Adj β^g^ (95% CI)							
Intuitive eating^h^	0.00 (ref)	−0.1 (−0.5, 0.5)	0.00 (ref)	0.7 (−0.2, 1.6)	−0.2 (−1.0, 0.6)	−0.2 (−1.0, 0.6)	−1.7 (−3.3, −0.1)

Racial minority or other includes American Indian, Alaskan Native, Native Hawaiian, Pacific Islanders. Models examining sex differences in disordered eating behaviors and intuitive eating were adjusted for age, ethnicity/race, parental maximum educational attainment, and BMI. Models examining ethnic/racial differences in disordered eating behaviors and intuitive eating were adjusted for age, sex, parental maximum educational attainment, and BMI. ^a^aPR = adjusted prevalence ratio. ^b–f^Disordered eating was assessed from the Questionnaire on Eating and Weight Patterns-5 (QEWP-5). ^d^Weight and shape concerns refer to weight and shape being the main things or most important. ^e^Associated symptoms of binge eating include: eating much more rapidly than usual, eating until uncomfortably full, eating large amounts of food when not feeling physically hungry, eating alone because embarrassed by how much you were eating, feeling disgusted with oneself, depressed, or feeling guilty after overeating. ^f^Unhealthy weight control behaviors were confirmed if participants responded yes to any of the unhealthy weight control behaviors assessed. ^g^adj β = adjusted beta coefficient, adjusted for age, ethnicity/race, parental education attainment, and BMI. ^h^Intuitive eating was assessed from the subscale of reliance on hunger and satiety from the Intuitive Eating Scales-2; higher scores of reliance on hunger and satiety intuitive eating indicate greater reliance on hunger and satiety intuitive eating.

#### Intuitive eating

The summary score of reliance on hunger and satiety intuitive eating for both male and female college students is reported in Appendix Table 1. The parameter estimates of the mean score difference of the reliance on hunger and satiety intuitive eating between male and female college students were close to the null value [adj β = −0.1 (95% CI = −0.5, 0.5)] after adjustment for sociodemographic variables and BMI (Table 2).

### Ethnic/racial differences in eating behaviors

#### Disordered eating behaviors

The prevalence of disordered eating behaviors across ethnic/racial groups is reported in Appendix Table 1. As shown in Table 2, compared to the non-Hispanic Whites, all ethnic/racial minorities had greater prevalence of associated symptoms of binge eating (aPR range = 1.4–2.3), although some 95% CIs included the null value after adjustment for age, sex, parental maximum educational attainment, and BMI. Additionally, non-Hispanic Black or African Americans and Hispanics each had 1.5 and 1.4 times the prevalence of concerns with weight and body shape (95% CI = 1.1–1.9 and 95% CI = 1.1–1.8 for non-Hispanic Black or African American and Hispanics, respectively) after adjustment for age, sex, parental maximum educational attainment, and BMI. No significant ethnic/racial differences in disordered eating behaviors were observed among ethnic/racial groups (Table 2).

#### Intuitive eating

The summary score of reliance on hunger and satiety intuitive eating across ethnic/racial groups is reported in Appendix Table 1. Compared to non-Hispanic White college students, the mean score of reliance on hunger and satiety intuitive eating was higher among non-Hispanic Black or African Americans (adj β = 0.7, 95% CI = −0.2, 1.6), whereas the reliance on hunger and satiety intuitive eating score was lower in racial minorities or others (i.e., American Indians, Alaskan Native, Native Hawaiian, Pacific Islanders, multiple racial identities) individuals after adjustment for sociodemographic variables and BMI (Table 2). The reliance on hunger and satiety intuitive eating score of Hispanics or non-Hispanic Asians was close to that of non-Hispanic Whites (adj β = −0.2, 95% CI = −1.0, 0.6 for both Hispanics and non-Hispanic Asians) (Table 2).

## Discussion

The present study aimed to examine sex and ethnic/racial differences in disordered eating behaviors and intuitive eating scores among a sample of ethnically/racially diverse college students in Texas. In this study, we observed both ethnic/racial and sex differences in disordered eating behaviors, but only ethnic/racial differences in the summary scores in intuitive eating cues.

The finding that overeating was more prevalent among male than female college students largely agrees with past results showing that overeating is prevalent among adolescent and young males (Cain et al., 2012; Sonneville et al., 2013; Yoon et al., 2022a). The greater prevalence of overeating among male college students found in our study confirms past findings and contributes to the substantial prevalence of overeating among adolescents, college students, and young males in general and may further indicate that such behaviors, particularly among males, may persist over time. As evidenced in previous research, societal pressure to demonstrate strength and conform to cultural notions of masculinity (Lavender et al., 2017; Stewardson et al., 2020; Goode et al., 2021; Stanaland and Gaither, 2021) may result in overeating behaviors as males strive to fulfill their caloric requirements for muscle growth or adhere to specific dietary patterns linked to developing muscle mass. Moreover, hormonal differences between males and females may also impact appetite regulation. For instance, testosterone, which is positively correlated with ghrelin, an appetite-regulating hormone, may potentially contribute to increased appetite and a tendency to overeat (Greenman et al., 2009).

Among female college students, binge eating and unhealthy weight control behaviors were more prevalent than among males, which aligns with past studies (Kelly-Weeder, 2012; Lee-Winn et al., 2014, 2016; Simone et al., 2022). Our results thus contribute to the literature by showing that the prevalence of associated symptoms of binge eating, a less-explored type of disordered eating behavior, is greater among female college students than among male college students (Lee-Winn et al., 2014). The greater prevalence of associated symptoms of binge eating, concerns about weight and body shape, and engagement in unhealthy weight control behaviors among female versus male college students collectively indicate that such students may perceive greater cultural pressure to achieve body idealization (e.g., pursue thinness), develop anxiety related to eating and/or food, and/or ultimately engage in such disordered eating behaviors to control their body weight. Thus, health professionals should consider educating female college students about how to nurture a healthy, positive understanding of their body shape and appearance to reduce, if not prevent, associated symptoms of binge eating. As an effort to prevent individuals from developing excessive concerns about weight, body shape, and appearance and from engaging in unhealthy weight control behaviors, preventive interventions exist, including the Body Project (National Eating Disorders Associations, 2022) and Student Bodies (Taylor et al., 2006). These programs provide valuable insights into how societal ideals of appearance and unrealistic standards of beauty influence the development of disordered eating behaviors and should be shared as a resource for college students. Recent empirical studies have shown that the Body Project has been effective in preventing eating disorders by reducing thin-ideal internalization, body dissatisfaction, dieting, and eating disorder symptoms across ethnic/racial groups of college students (Stice et al., 2014, 2021). These findings indicate that such interventions and programs may play an active role in promoting body positivity and equipping students with the necessary tools to navigate societal pressures and maintain healthy relationships with their bodies.

The lack of statistically significant sex differences in the summary scores for the cues in intuitive eating also contributes to the growing body of literature on intuitive eating. The mentioned lack of significant sex differences conflicts with past results concerning college students showing that males had higher levels of intuitive eating scores than females (Tylka and Kroon Van Diest, 2013; Rodgers et al., 2021). The nonsignificant sex difference in scores for cues in intuitive eating may indicate the need for sex-inclusive programs to be developed as a means to increase awareness about intuitive eating and drive engagement in intuitive eating, particularly by encouraging the practice of relying on hunger and satiety cues when eating.

Ethnic/racial differences in eating behaviors emerged in our study as well. Among the college students, most disordered eating behaviors were more prevalent among non-Hispanic Black or African Americans, Hispanics, non-Hispanic Asians, and racial minorities and others (i.e., American Indians, Alaskan Natives, Native Hawaiians, Pacific Islanders, and multiple racial identities) than among non-Hispanic White college students. In particular, associated symptoms of binge eating were more prevalent among non-Hispanic Black or African American, Hispanic, and non-Hispanic Asian college students than their non-Hispanic White peers. This result suggests that ethnic/racial minorities may face greater challenges and difficulties in recognizing internal sensations and cues related to hunger and fullness (Piran, 2016; Niva, 2017). Difficulties in accurately perceiving those internal cues may lead individuals to override or ignore hunger cues, thereby resulting in their reliance on external stimuli and potentially contributing to the development of disordered eating behaviors (Assari, 2018; Gattario and Frisén, 2019). Moreover, African American college students in the United States may face pressure to conform to Western cultural and societal standards of beauty as well as cultural ideals of thinness as the ideal body type (Awad et al., 2015), which contrasts with the societal standards and cultural ideals of African American culture that encourage the pursuit of larger body frames. Because the contrasting standards of beauty may lead African Americans to engage in disordered eating behaviors, future studies should examine acculturation and bicultural stress among ethnic/racial minorities and how such stressors and distress may relate to disordered eating behaviors.

Likewise, Hispanic college students, especially those exposed to Western media and societal beauty standards may experience conflicts between cultural ideals and external pressures for thinness given the Hispanic culture’s strong emphasis on family, community, and traditional food practices (Romo et al., 2015). This conflict, may contribute to body dissatisfaction and disordered eating behaviors. Overall, the greater prevalence of disordered eating behaviors among ethnic/racial minorities compared to non-Hispanic Whites stresses the importance of further examining cultural influences on eating behaviors. Considering the potential influence of cultural ideals on body dissatisfaction and subsequent engagement in disordered eating behaviors, future studies should consider examining whether ethnic/racial-minority college students experience greater pressure to conform to prevailing beauty and/or body standards compared to non-Hispanic White college students. Although immigration status was not specifically assessed in our survey, future studies should additionally explore and evaluate the impact of acculturation and bicultural stress on eating behaviors among Hispanic students. Striking a balance between traditional cultural values and the values of one’s new culture may create tension and confusion, which can manifest in disordered eating behaviors as individuals strive to fit in or cope with conflicts related to their cultural identity. Examining those dynamics can provide valuable insights into the interplay between cultural factors and disordered eating behaviors in ethnic/racial populations.

In the various ethnic/racial groups that we examined, non-Hispanic Black or African Americans were the sole ethnic/racial group with significantly higher summary scores for cues in intuitive eating than non-Hispanic White college students. This finding suggests that non-Hispanic Black or African Americans may demonstrate a reduced ability to recognize internal sensations and cues associated with hunger and fullness, which may partly account for their higher summary scores. This finding aligns with past findings showing that African Americans were more likely to report engaging in intuitive eating, as assessed by a single item, than non-Hispanic White individuals (Denny et al., 2013). Higher summary scores for the cues in intuitive eating among non-Hispanic Black or African American than non-Hispanic White college students may indicate that the cultural value of food in African American culture (e.g., connection, celebration, and comfort) may partly align with the principles of intuitive eating, which emphasizes attunement to one’s body and the enjoyment of eating food. Moreover, higher summary scores for the cues in intuitive eating observed among non-Hispanic Black or African Americans in our study support the growing movement within their communities that promotes body positivity, self-acceptance, and the rejection of diet cultures (Cwynar-Horta, 2016). Such movements emphasize embracing diverse body shapes and sizes, challenging society’s ideals of beauty, and encouraging individuals to listen to their bodies instead of focusing solely on weight and/or appearance (Cwynar-Horta, 2016). Along similar lines, the greater summary scores for the cues in intuitive eating among non-Hispanic Black or African Americans align with studies documenting the group’s possession of interoceptive awareness skills (Price and Hooven, 2018) and heightened sensitivity to physical hunger cues and satiety signals (Barraclough et al., 2019). Although non-Hispanic Black or African Americans may have higher rates of obesity (Sa et al., 2016), higher summary scores among non-Hispanic Black or African Americans in our study and others show that intuitive eating is effective in weight management (Hawks et al., 2005). Thus, caution should be exercised in the implementation of preventive efforts and strategies to address obesity in that population, so as not to inadvertently discourage intuitive eating. In a broader context, because food insecurity and insufficient access to food are significant dilemmas among non-Hispanic Black or African American college students and are associated with obesity (Goode et al., 2021), future studies should further consider examining food insecurity in conjunction with intuitive eating.

The strengths of the present study include the assessment of eating behaviors among a diverse sample of college students, all of whom are in the transitional period between adolescence and young adulthood. Unlike studies on eating behaviors that have focused on women and non-Hispanic White individuals (Kelly-Weeder, 2012; Lundahl et al., 2015; Phillips et al., 2016; Smith et al., 2020; Gödde et al., 2022), this study was conducted in a Hispanic- and Asian-serving institution. Thus, the number of males and females as well as the number of ethnically/racially diverse participants were large and roughly equal, which allowed us to present results stratified by sex and ethnicity/race and have many individuals in each subgroup.

Despite those strengths, our study also had limitations. First, participants formed a nonrandomly selected convenience sample of primarily Hispanic and non-Hispanic Asian college students enrolled in an introductory course in kinesiology at a Hispanic- and Asian-serving institution. Therefore, the study’s results may not represent all college students in the United States. The course, Introduction to Kinesiology, falls within the Texas core curriculum and is thus open to enrollment for all undergraduate students irrespective of their major. However, importantly, individuals with a stronger interest in their bodies may have consciously opted to enroll in this course over alternative courses. Moreover, research has demonstrated that kinesiology undergraduate students tend to display higher levels of body awareness, aversion to weight gain, weight-related bias, and negative attitudes toward weight (Alberga et al., 2016; Wijayatunga et al., 2019). Consequently, it is plausible that this study attracted participants who had heightened body consciousness and a greater awareness of their eating habits. Furthermore, using a nonprobability convenience sampling approach and recruiting students from a single campus and specific course within a single semester may have introduced cohort effects and generated selection bias, which may have influenced the study’s results. Beyond that, participants were recruited from Houston, Texas, which limits the findings’ generalizability to geographical regions within the southern United States. For these reasons, the study findings are subject to bias, necessitating further examination with nationally representative samples to replicate the study results.

Second, due to the small number of participants who self-identified as American Indian, Alaskan Native, Native Hawaiian, Pacific Islander, or multiple racial identities or other, those participants were grouped into a single category: “racial minority or other.” Given the group’s small sample size (*n* = 24) and point estimates accompanied by wide confidence intervals, lower summary scores for the cues in intuitive eating among the group’s members than among non-Hispanic White college students should be interpreted with caution. Future studies should also examine the eating behaviors of understudied ethnic/racial groups (Vigil et al., 2021). Moreover, our survey did not gather information regarding Hispanic or Asian origin and therefore did not distinguish cultural subgroups of Latin Americans from Asians. Third, in this study, sex referred to sex assigned at birth. Eating behaviors among gender minorities were not examined because the survey did not question participants about their current gender. Thus, our findings may not be generalizable to transgender, nonbinary, genderfluid, or intersex populations (Nagata et al., 2020). Our choice to not assess gender prevented us from exploring the intersectionality between gender and ethnicity/race on eating behaviors. Fourth, for intuitive eating, other subscales besides reliance on hunger and satiety cues were not assessed. However, we chose the reliance on hunger and satiety cues subscale as a proxy of intuitive eating because it captures one of the most distinct differences between disordered eating behaviors and intuitive eating: disordered eating behaviors rely on external factors, whereas intuitive eating emphasizes reliance on internal hunger cues when eating. Fifth, using self-report data may have underestimated the prevalence of socially unacceptable behaviors. Therefore, participants may have reported socially desirable behaviors, which may have over- or underestimated the findings. In a related context, the term “overeating” in this study was intended to denote the consumption of substantial food quantities outside of special occasions or events. However, the definition of “overeating” conformed to DSM-5, which defines “overeating” as “eating in a discrete period of time (e.g., within any 2-h period), and the amount of food that is definitely larger than what most people eat in a similar period of time under similar circumstances. Since further contextual details of overeating were not explicitly stated, it might have resulted in subjective interpretations of “overeating.” Therefore, caution is required when interpreting the findings related to “overeating.” Sixth, given our study’s cross-sectional design with data collected at only one time point, causal inferences among variables should not be drawn. Finally, as in all observational studies, our study was also limited by potential residual confounding from unmeasured or unknown confounders.

Our findings have important implications for research. The substantial prevalence of disordered eating behaviors across sex and ethnicity/race categories reported in our study illustrates the need to better understand disordered eating behaviors among men and ethnic/racial minorities, groups that have historically been neglected in the literature on eating behaviors (Lavender et al., 2010; Cain et al., 2012). The nonsignificant difference in summary scores for the cues in intuitive eating across the sexes may illustrate the need for sex-inclusive research to examine the cues in intuitive eating among college students and young adults (Denny et al., 2013).

Our findings may also have implications for practitioners, particularly for clinicians who work closely with college students who engage in disordered eating and rely less on hunger and satiety cues in intuitive eating. The substantial prevalence of disordered eating among college students and the greater prevalence of disordered eating in certain sex-based and ethnic/racial groups may provide insights into building awareness about sex-based and ethnic/racial disparities in the cues in intuitive eating among college students. The nonsignificant differences in mean scores for cues in intuitive eating between female and male college students and among non-Hispanic Asian, Hispanic, and non-Hispanic White college students imply that inclusive support for college students to continue to eat more intuitively and mindfully may ensure that health equity is reached across all college students, regardless of sex and ethnicity/race.

## Conclusion

In our sample of college students, the prevalence of disordered eating behaviors, including binge eating, unhealthy weight control behaviors, associated symptoms of binge eating, and concerns with weight and body shape, was greater among females and ethnic/racial minorities than among males and non-Hispanic White individuals. The mean summary score for the cues in intuitive eating was higher among non-Hispanic Black or African Americans than among non-Hispanic White college students, whereas the scores among other ethnic/racial groups and the sexes did not differ. The dearth of studies that have examined sex and ethnic/racial disparities in the cues in intuitive eating calls for additional replication studies investigating those differences.

## Data availability statement

The raw data supporting the conclusions of this article will be made available by the authors, without undue reservation.

## Ethics statement

The studies involving humans were approved by University of Houston IRB Board. The studies were conducted in accordance with the local legislation and institutional requirements. The participants provided their written informed consent to participate in this study.

## Author contributions

CY, TL, RB, and CJ designed the study and collected the data. CY defined the hypotheses, analyzed the data, and wrote the first draft with contributions from DM, KK, TL, RB, and CJ. All authors reviewed and commented on subsequent drafts of the manuscript.

## Conflict of interest

The authors declare that the research was conducted in the absence of any commercial or financial relationships that could be construed as a potential conflict of interest.

## Publisher’s note

All claims expressed in this article are solely those of the authors and do not necessarily represent those of their affiliated organizations, or those of the publisher, the editors and the reviewers. Any product that may be evaluated in this article, or claim that may be made by its manufacturer, is not guaranteed or endorsed by the publisher.

## Footnotes

^1^ www.qualtrics.com

## Appendix 1

Prevalence of disordered eating and mean scores of reliance on hunger and satiety intuitive eating (*N* = 887).

**Table:**

	Sex			Ethnicity/race					
	Male (*n* = 406)	Female (*n* = 481)	*p* value	Non-Hispanic White (*n* = 134)	Non-Hispanic Black or African American (*n* = 125)	Hispanic (*n* = 294)	Non-Hispanic Asian American (*n* = 310)	Racial- minority or multiracial (*n* = 24)	*p* value
Disordered eating behaviors aPR^a^ (95% CI)									
Overeating^b^	49.3 (49.1, 49.5)	41.6 (41.4, 41.8)	.02	44.0 (43.6, 44.4)	48.0 (47.6, 48.4)	47.3 (47.1, 47.5)	41.3 (41.1, 41.5)	58.3 (57.5, 59.1)	0.3
Binge eating^c^	32.0 (31.7, 32.3)	58.0 (57.7, 58.3)	<.01	47.5 (47.0, 48.0)	38.3 (37.8, 38.8)	43.9 (43.5, 44.3)	50.0 (49.6 50.4)	28.6 (27.4, 29.8)	0.4
Unhealthy weight control behaviors^d^	44.6 (44.4, 44.8)	58.2 (58.0, 58.4)	<.01	44.8 (44.4, 45.2)	47.2 (46.8, 47.6)	51.4 (51.2, 51.6)	57.7 (57.5, 57.9)	50.0 (49.2, 50.8)	0.09
Associated symptoms of binge eating^e^	22.9 (22.7, 23.1)	35.1 (34.9, 35.3)	<.01	30.6 (30.2, 31.0)	15.2 (14.7, 15.7)	33.7 (33.4, 34.0)	31.6 (31.4, 31.8)	20.8 (19.8, 21.8)	<0.01
Concerns with weight and body shape^f^	40.6 (40.4, 40.8)	51.6 (51.4, 51.8)	<.01	42.5 (42.1, 42.9)	34.4 (34.0, 34.8)	52.4 (52.2, 52.6)	48.1 (47.9, 48.3)	41.7 (40.9, 42.5)	0.01
Intuitive eating Adj β^g^ (95% CI)									
Reliance on hunger and satiety intuitive eating^h^	18.5 (18.1, 18.9)	18.4 (18.0, 18.8)	0.9	18.5 (17.7, 19.3)	19.3 (18.5, 20.1)	18.3 (17.9, 18.7)	18.4 (18.0, 18.8)	16.9 (16.5, 17.3)	0.04

Racial minority or other includes American Indian, Alaskan Native, Native Hawaiian, Pacific Islanders. Models examining sex differences in disordered eating behaviors and reliance on hunger and satiety intuitive eating were adjusted for age, ethnicity/race, parental maximum educational attainment, and BMI. Models examining ethnic/racial differences in disordered eating behaviors and reliance on hunger and satiety intuitive eating were adjusted for age, sex, parental maximum educational attainment, and BMI. ^a^aPR, adjusted prevalence ratio. ^b–f^Disordered eating was assessed from the Questionnaire on Eating and Weight Patterns-5 (QEWP-5). ^d^Weight and shape concerns refer to weight and shape being the main things or most important. ^e^Associated symptoms of binge eating include: eating much more rapidly than usual, eating until uncomfortably full, eating large amounts of food when not feeling physically hungry, eating alone because embarrassed by how much you were eating, feeling disgusted with oneself, depressed, or feeling guilty after overeating. ^f^Unhealthy weight control behaviors were confirmed if participants responded yes to any of the unhealthy weight control behaviors assessed. ^g^adj β = adjusted beta coefficient, adjusted for age, ethnicity/race, parental education attainment, and BMI. ^h^Reliance on hunger and satiety intuitive eating was assessed from the subscale of reliance on hunger and satiety from the Intuitive Eating Scales-2; higher scores of reliance on hunger and satiety intuitive eating indicate greater reliance on hunger and satiety intuitive eating.
